# Predictors of cognitive enhancement after training in preschoolers from diverse socioeconomic backgrounds

**DOI:** 10.3389/fpsyg.2014.00205

**Published:** 2014-03-13

**Authors:** M. Soledad Segretin, Sebastián J. Lipina, M. Julia Hermida, Tiffany D. Sheffield, Jennifer M. Nelson, Kimberly A. Espy, Jorge A. Colombo

**Affiliations:** ^1^Unidad de Neurobiología Aplicada, Consejo Nacional de Investigaciones Científicas y Técnicas (CONICET), Centro de Educación Médica e Investigaciones Clínicas Norberto Quirno (CEMIC), Ciudad Autonoma de Buenos AiresBuenos Aires, Argentina; ^2^Office of Research, University of NebraskaLincoln, NE, USA; ^3^Department of Psychology, University of NebraskaLincoln, NE, USA; ^4^Department of Psychology, University of OregonEugene, OR, USA

**Keywords:** cognitive development, intervention, SES, mixed models, socio-environmental predictors, preschool children

## Abstract

The association between socioeconomic status and child cognitive development, and the positive impact of interventions aimed at optimizing cognitive performance, are well-documented. However, few studies have examined how specific socio-environmental factors may moderate the impact of cognitive interventions among poor children. In the present study, we examined how such factors predicted cognitive trajectories during the preschool years, in two samples of children from Argentina, who participated in two cognitive training programs (CTPs) between the years 2002 and 2005: the *School Intervention Program* (SIP; *N* = 745) and the *Cognitive Training Program* (CTP; *N* = 333). In both programs children were trained weekly for 16 weeks and tested before and after the intervention using a battery of tasks assessing several cognitive control processes (attention, inhibitory control, working memory, flexibility and planning). After applying mixed model analyses, we identified sets of socio-environmental predictors that were associated with higher levels of pre-intervention cognitive control performance and with increased improvement in cognitive control from pre- to post-intervention. Child age, housing conditions, social resources, parental occupation and family composition were associated with performance in specific cognitive domains at baseline. Housing conditions, social resources, parental occupation, family composition, maternal physical health, age, group (intervention/control) and the number of training sessions were related to improvements in specific cognitive skills from pre- to post-training.

## Introduction

Broadly defined, executive functions (EF) refer to a complex set of cognitive abilities that underlie adaptive, goal-directed behaviors, and enable individuals to override more automatic or established thoughts and responses (Garon et al., 2008; Diamond, 2013). EF are critical when solving novel problems and thus essential for self-regulation, school learning, and social behavior (e.g., Hughes and Graham, 2002; Anderson, 2002; Isquith et al., 2005; Diamond et al., 2007; Garon et al., 2008; Bull et al., 2011; Espy et al., 2011). At a more fine-grained level a set of cognitive control skills (e.g., attention, inhibitory control, self-monitoring, and flexibility) is defined as specific interrelated information-processing abilities that are involved in the control and coordination of information in the service of goal-directed actions, as studied in the cognitive development literature (Willoughby et al., 2012). Focusing on these more narrowly defined abilities is particularly suitable when studying EF in early childhood, as many of the more complex aspects of EF (e.g., abstract thought; goal setting) have an extended developmental course and are not easily measured in very young children (Garon et al., 2008; Willoughby et al., 2012). The emergence and development of those cognitive processes depend on both biological maturation and environmental experiences (Fisher, 2006; Berkman et al., 2012), and follow different trajectories from the first year of life (Anderson, 2002; Garon et al., 2008; Marsh et al., 2010; Howe et al., 2012). In addition, these trajectories are sensitive to individual differences, and the quality of the micro- and mesosystemic developmental contexts (home and school) (Lipina and Colombo, 2009; Cadima et al., 2010; Sarsour et al., 2011).

Several studies have suggested that associations between socioeconomic status and cognitive development during childhood are mediated by biological, psychological and environmental factors, which may be conceptualized at multiple levels of analysis (individual, family, and social contexts), and increase the likelihood of negative impacts later in life (Leinonen et al., 2002; Raver et al., 2007, 2013; Santos et al., 2008; Cadima et al., 2010; Rhoades et al., 2011; Sarsour et al., 2011; Lipina et al., 2013). Among the environmental factors that have been associated with these impacts, the following are the most cited in the scientific literature: family income, family composition, parental level of education and occupation, housing conditions, perinatal health factors, quality of home and school environments, attendance to early education programs, parental mental health, parenting styles, parent-child interactions, neighborhood characteristics, and social support (Burchinal et al., 2000; Bradley and Corwyn, 2002, 2005; Evans, 2004; Gassman-Pines and Yoshikawa, 2006; Engle et al., 2007; Grantham-McGregor et al., 2007; Walker et al., 2007; Rhoades et al., 2011; Sarsour et al., 2011). Additionally, the impact of these factors on different aspects of child development may vary according to the type, number and accumulation of risk factors to which children are exposed, the timing of exposure, and the individual susceptibility to each one (Najman et al., 2004; Stanton-Chapman et al., 2004; Belsky et al., 2007; Walker et al., 2007; Kiernan and Huerta, 2008; Flouri et al., 2009; Kiernan and Mensah, 2009; Hall et al., 2010; Rhoades et al., 2011; Evans et al., 2013). Thus, it is not only the mere presence or absence of specific risk factors that influence development, but also their accumulation in a context of individuality, with more risk leading to greater adjustment difficulties (Burchinal et al., 2000; Stanton-Chapman et al., 2004; Appleyard et al., 2005; Gassman-Pines and Yoshikawa, 2006; Cadima et al., 2010; Evans et al., 2013). Despite the significant advances in the field, more research is necessary to elucidate specific environmental experiences that contribute to individual differences in cognitive control development (Rhoades et al., 2011), as well as their contribution to individual differences in the context of intervention trials.

Specifically, cognitive control performance of children living in poverty is limited in its potential due to the presence of multiple risk factors in these contexts, such as child health history (peri- and postnatal), maternal education, parental mental health, quality of stimulation at home, and social interactions in different contexts (e.g., home and school). Results from studies developed in Argentina assessing associations between poverty and impact on cognitive processing have verified the modulation of different cognitive processes (i.e., attentional, inhibitory control, working memory, flexibility, and planning) in infants and preschoolers as a result of socioeconomic status and income, as well as the influence of poverty on academic performance (i.e., language and mathematics) in elementary and high school children (Lipina et al., 2004, 2005, 2013; Segretin et al., 2009).

In Argentina, according to the latest data published by the National Institute of Statistics and Censuses (INDEC, 2001), 14.3% of families live with Unsatisfied Basic Needs (UBN). Additionally, the Observatory on the Argentinean Social Debt reported that 29.6% of the population was poor during 2010 (Tuñón, 2011). With respect to child poverty, 40.5% of children under the age of 14 were living in poverty, and 14.2% in extreme poverty in 2006 (INDEC, 2006). According to the report of the United Nations Fund for Children of 2010, 28.7% of children under the age of 18 in Argentina are in poverty (CEPAL-UNICEF, 2010).

During the past decade, several interventions targeting cognitive control development have been designed and evaluated in the fields of developmental psychology and developmental cognitive neuroscience (Lipina and Colombo, 2009; Burger, 2010). The main goals of such interventions were the promotion of cognitive control development in early childhood, with the aim of influencing broader, long-term outcomes, such as academic and social adjustment (McCandliss et al., 2003; Temple et al., 2003; Colombo and Lipina, 2005; Klingberg et al., 2005; Rueda et al., 2005; Wilson et al., 2006; Diamond et al., 2007; Stevens et al., 2008; Beatty, 2009; Thorell et al., 2009; Barnett, 2011; Espinet et al., 2012). Most of the previous interventions were successful in promoting cognitive performance in the short- or medium-term, and evaluation of their success generally focused on pre- and post-training performance comparisons between groups. Only few studies have also included an analysis of the predictors of intervention impact, mostly based on variables, such as the initial cognitive performance, age, and/or program characteristics (e.g., Bierman et al., 2008). The effectiveness of the intervention programs that include cognitive stimulation modules has been related to the following aspects of program design: (a) comprehensiveness of services (educational, nutritional, sanitation, and social services); (b) teacher and family participation; (c) direct and indirect interventions; (d) quality of services; (e) staff recruitment and training; and (f) cultural pertinence of interventions (Ramey and Ramey, 1998, 2003; Gray and McCormick, 2005; Karoly et al., 2005; Reynolds and Temple, 2005; Perez-Johnson and Maynard, 2007; Barnett, 2011; Reynolds et al., 2011). It is important, however, to consider that interventions are not equally effective for all participants. In this regard, different factors could moderate the impact of the intervention, and this information would be crucial to the design of new interventions, both experimental and applied.

In general, studies on the moderation of cognitive development by environmental factors have focused on the associations between child poverty and accumulation of risk factors (Gassman-Pines and Yoshikawa, 2006; Weiland and Yoshikawa, 2012; Flouri et al., 2013; Meunier et al., 2013). Less analytical efforts have been devoted to moderation based on risk factors in the area of intervention science. Thus, the implementation of risk-factor analysis, such as identifying different socio-environmental variables as predictors of intervention impact, is important in order to establish targets for improvement in the design of innovative interventions.

In this article, we propose the analysis of specific aspects of two intervention programs implemented in Argentina between 2002 and 2005, with the main goal of optimizing cognitive control performance in preschool children: the *School Intervention Program* (SIP), and the *Cognitive Training Program* (CTP) (Table 1). We focus the analysis on the identification of different socio-environmental predictors of cognitive trajectories. Specifically, the main goals of the present study were: (1) to examine how environmental factors moderate cognitive performance; and (2) to identify factors that moderate the impact of two intervention programs aimed at optimizing cognitive performance in two samples of poor- and non-poor preschoolers. We examined children's performance in tasks demanding attention, inhibitory control, memory, flexibility, and planning, and considered the impact of environmental risk factors on each cognitive task at baseline and task trajectories (change in performance from pre- to post-assessment). Mixed model analyses were applied in order to identify socio-environmental predictors associated with higher levels of cognitive control performance in the pre-intervention phase, and with increased improvement in cognitive control between pre- and post-intervention phases. We expected that higher cognitive performance at baseline would be associated with better socio-environmental conditions (Feldman and Eidelman, 2009; Kiernan and Mensah, 2009). Additionally, we expected that children living in families with more resources (in terms of parental occupation and education, financial resources, type of housing and social support) would have higher improvements in their cognitive performance after training. This hypothesis was based on the idea that for children from worst socio-environmental conditions another type of intervention would be required (e.g., more specific for each cognitive skill, with more intervention intensity exposure-considering frequency, length, and age) (Ramey and Ramey, 2003). We also expected to identify different predictors for each cognitive process and program, taking into account the differences in the cognitive developmental trajectories at these ages (Garon et al., 2008), and the differences in the program characteristics (e.g., context of implementation, number and frequency of training sessions, and modalities of training) (Jolles and Crone, 2012). Also, it is important to mention that results from different intervention programs for disadvantaged children have suggested that the frequency of intervention is a significant modulator of the impact (Ramey and Ramey, 2003; Karoly et al., 2005; Burger, 2010). Based on this, we also expected that the number of training sessions (exposure to training) would be a significant predictor of cognitive trajectories, with higher cognitive performance improvements in children with more exposure to training.

**Table 1:** **Programs descriptions**.

	**Program**	
	**SIP**	**CTP**
Design	Experimental, controlled, and random study.	Quasi-experimental and random study.
Participants	Children from 3 to 5 years from UBN^a^ homes. City of Buenos Aires.	Children from 3 to 5 years from UBN and SBN^b^ homes. City of Salta.
Study groups	Intervention/control.	Individual training modality/group training modality^*^.
Program phases	1. Cognitive assessment (Time 1)	1. Cognitive assessment (Time 1)
	2. Intervention modules implementation	2. Intervention modules implementation
	3. Cognitive assessment (Time 2)	3. Cognitive assessment (Time 2)
Intervention modules	Cognitive training; nutritional supplementation; counseling for parents; training; and counseling for teachers.	Cognitive training; nutritional supplementation (the government agency provided counseling for parents and adults working in the childcare centers).
Activities for the cognitive training module	Exercising activities (individual modality of training).	Activities with a pedagogical format (individual and group modalities of training).
Frequency of intervention	Once a week for 16 weeks in 1 year; twice a week during 16 weeks in 1 year.	Twice a week for 16 weeks in 1 year.
Total cognitive training sessions	16 or 32 sessions.	32 sessions.
Context of implementation	Kindergartens.	Childcare Centers.

* Only 4-year-old children were randomly assigned to individual or group training modalities. For that reason, analysis were run separately for this age group (with the aim to compare training modalities) (manuscript under revision), and in the present article only 3- to 5-year-old children assigned to the group training modality were considered for the prediction analysis.

a UBN (poverty criteria, see details in section Socioeconomic, Life, and Health Condition Measures).

b SBN: Satisfied Basic Needs.

## Methods

### Study design, participants, and procedures

#### SIP program

A sample of healthy Argentinean children aged 3–5 years participated in the SIP, a longitudinal study implemented between years 2002 and 2004 in three kindergartens in the city of Buenos Aires, selected by applying the conglomerate sample method. The program was an experimental, randomized and controlled study with the main goal to train cognitive performance in preschool children from UBN homes (Colombo and Lipina, 2005; Lipina et al., 2012). Seven hundred and forty five preschool children were authorized to participate. We have verified an attrition rate around 15% per year. Each year new cohorts were enrolled, forming different study groups with 1 and 2 years of intervention. For the present article only data from children with 1 year of intervention between the years 2002 and 2004 were analyzed, because of the small sample size for 2 years of intervention, which did not allow executing the planned analytical procedures. Informed consents were obtained from parents/caregivers, and ethical approval was obtained from the Ethical Review Committee. The study was conducted according to APA's ethical standards, and international and national children rights laws.

Before the beginning of the program, we recruited and trained a group of college students (“trainers” from now on) from the school of psychology and education. During the same period, we informed the parents of children attending the selected institutions about the program activities and we asked them to sign written consents to include their children into the program. After that, from April to July in each year, we evaluated children's cognitive performance (Time 1, baseline) (see section Cognitive Measures), and parents attended individual interviews to give socioeconomic, sociodemographic and child health history information. Then, four intervention modules were implemented from July to November (see above). After the intervention, all children had a final cognitive assessment (Time 2, post-intervention) with the same battery of tasks used at Time 1. We provided trainers with an intervention procedure guide and supervised them daily. Trainers had to complete a form describing the implemented activities, which we revised daily to suggest adjustments. Trainers were blind to the hypotheses of the study. Activities for the control and intervention groups were organized in different days of the week and all trainers were reassigned to different schools for the final phase (post-intervention cognitive assessment).

**Figure 1:**
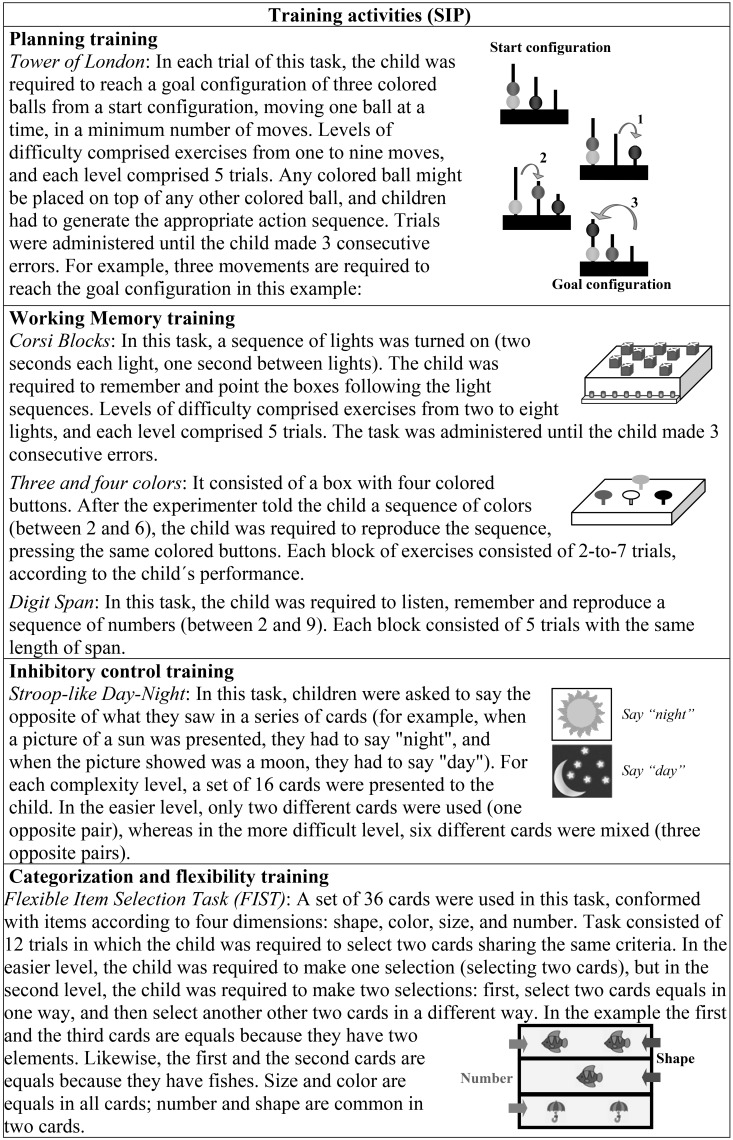
**Description of training activities in the SIP**.

The program included the following four modules:

(1) The individual *cognitive training module* consisted of different exercises demanding cognitive control, with increasing complexity. Activities involved some of the tasks used for the pre- and post-assessment, using different trials (Figure 1). Exercises were implemented during the school day as an extracurricular activity. Two schemes were applied: once a week (a total of 16 sessions), or twice a week (a total of 32 sessions) during 16 weeks in 1 year. Activities followed a scheme previously designed considering the cognitive demand and the time available for the session (30/40 min), and were implemented by one trainer (adults/children ratio = 1 per 1), who was the same for each child.

**Figure 2:**
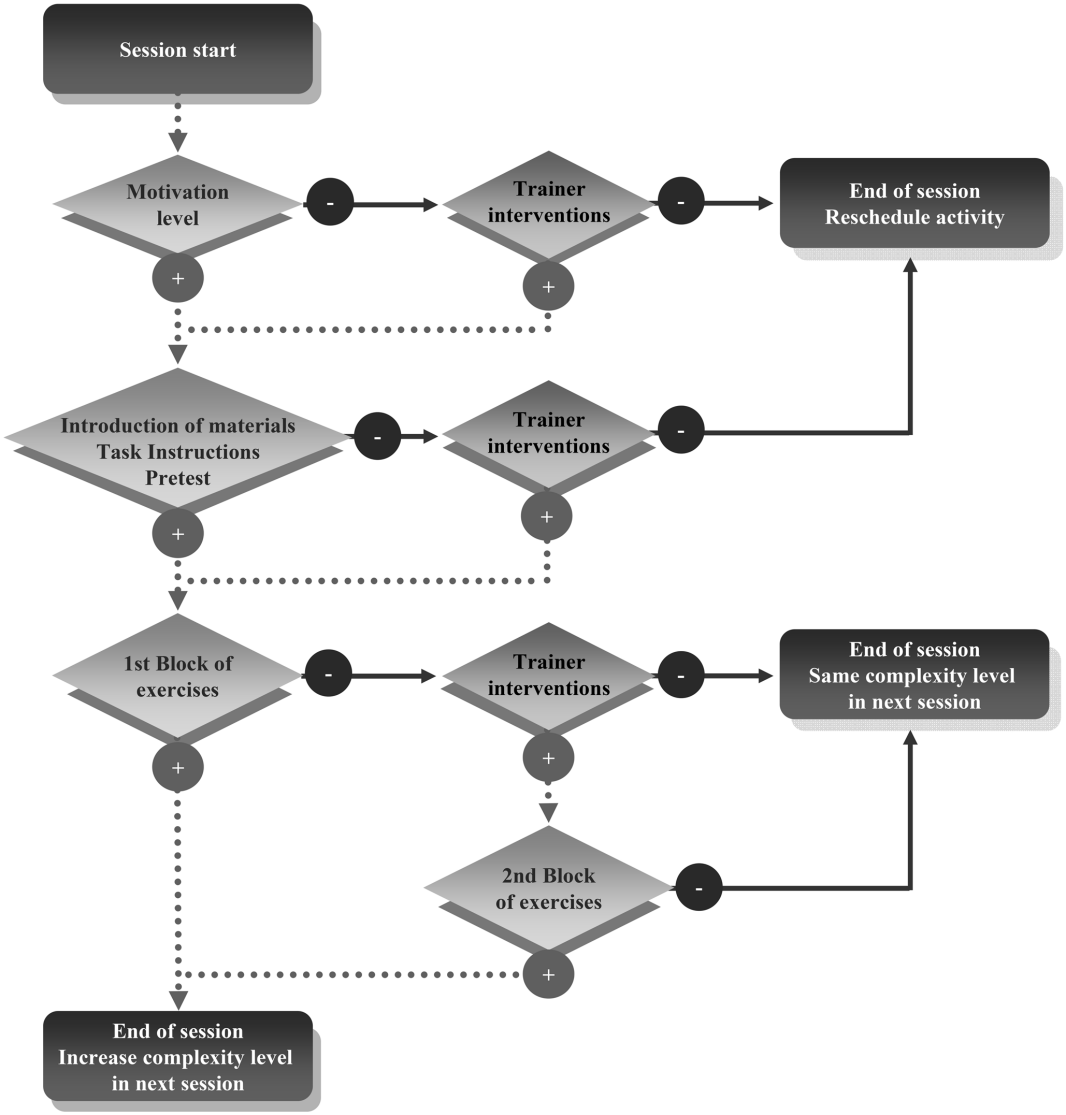
**Flowchart of the structure of each training session**.

Each training session was structured in four consecutive steps: (1) assessment of children's motivational state with a Likert scale including the following constructs: willing/not willing to collaborate, extroversion/introversion, talkative/quiet, active/passive, impulsive/thoughtful, trustful/distrustful; (2) introduction of novel materials for the activity and task instructions; (3) evaluation of instruction comprehension with pretest exercises; and (4) activities: blocks of different exercises or trials. Only when children were adequately motivated and pretests were properly solved, trainers continued with the next step, otherwise, the activity was scheduled for a new day in the same week. With respect to the fourth step, for each activity children were asked to solve different exercises organized in blocks of 5–10 trials (two blocks of exercises per session). After each block of exercises was completed, trainers evaluated children's performance, and determined the complexity level for the second block. When child efficiency in the first block of exercises reached at least 80%, the trainers increased the complexity level for the next block; otherwise, after trainers' intervention (according to the child's difficulties, considering the problem solving scheme), the second block of exercises presented new trials with the same level of difficulty. Performance on the last block of exercises determined the initial level of difficulty in the next session (Figure 2).

The theoretical framework of the cognitive training module was based on the problem-solving framework proposed by Zelazo et al. (1997). It involves four temporally and functionally distinct steps and substeps: problem representation, planning, execution, and evaluation (detection and correction). To solve a problem, first it is necessary to create or restructure the problem representation, including its possible solutions. Another component considered in the cognitive training module was the dynamic testing approach proposed by Sternberg and Grigorenko (2002). This form of testing proposes giving the children some kind of feedback in order to help them improve their scores, which in turn is based on Vigotsky's conception of the proximal development zone. Finally, two other components of the cognitive training module were the inclusion of challenge activities or trials, and repeated practice (Diamond, 2012).

(2) The *nutritional supplementation module* (implemented for both groups) consisted of the administration of one pill per week during the cognitive training period. Each pill contained 60 mg of elementary iron and 0.4 mg of folic acid, and was provided by UNICEF-Argentina.
(3) *Parental Counseling* was a module implemented for both groups, throughout the school year. Activities in this module included: (a) parent counseling; (b) child clinical exam; (c) child blood extraction to identify levels of hemoglobin; and (d) parent interviews.
(4) *Teacher training and counseling* (for both groups) was the fourth module, implemented throughout the school year, twice a month.

#### CTP program

Based on the results of the *SIP*, the same group of researchers designed a new CTP. In the year 2005, this program was implemented in the city of Salta in the context of a quasi-experimental prospective design (Segretin et al., 2007a,b; Lipina et al., 2012). Specifically, the aims of the program involved fostering cognitive development in preschoolers from UBN and Satisfied Basic Needs (SBN) homes with a reduction in the adult to child ratio (more children per adult) compared to the previous experience (SIP) (1/15 vs. 1/1, respectively). For this longitudinal study, a sample of 382 healthy Argentinean children aged 3–5 years were recruited from official childcare centers in the city of Salta in Argentina (Secretary of Children and Family from the Government of the Province of Salta) applying a conglomerate sample method. The rate of attrition was 15%. Informed consents were obtained from parents/caregivers, and ethical approval was obtained from the Ethical Review Committee. The study was conducted according to APA's ethical standards, and international and national children rights laws.

In this program, we recruited and trained a group of trainers, and we informed parents of children attending the selected institutions about the program activities and asked them to sign written consents to include their children into the program. After that, from April to July, we evaluated children's cognitive performance (Time 1) (see section Cognitive Measures), and parents attended individual interviews to give socioeconomic, sociodemographic and child health history information. Then, 4-year-old children were randomly assigned to an individual or group modality of cognitive training. Three- and 5-year-old children were all assigned to the group modality. The reasons for such design were: (1) authorities did not allow the research team to generate a control group for ethical reasons (i.e., they considered that all children had to receive the same activities, and that the government was not a research agency aimed at supporting research practices); and (2) authorities required to reduce the number of human resources for the execution of the program (i.e., individual training modality requires more trainers). For the present study, only children assigned to the group modality of training were analyzed (*n* = 333). The rest of the children (49) were trained with the individual modality, which was similar to the one implemented in the SIP (Colombo and Lipina, 2005; Lipina and Segretin, 2006; Martelli et al., 2007), to have a comparative training group (these children are not considered further in this paper) (Segretin et al., submitted for publication). Then, from July to November two intervention modules were implemented (see above), and after that, all children were administered a final cognitive assessment (Time 2) with the same battery of tasks used at Time 1. Like in the previous program, trainers' work in each phase was supervised daily during the year, and they were provided a procedure guide. Also, trainers had to complete a form for each activity, which were reviewed daily by supervisors.

**Figure 3:**
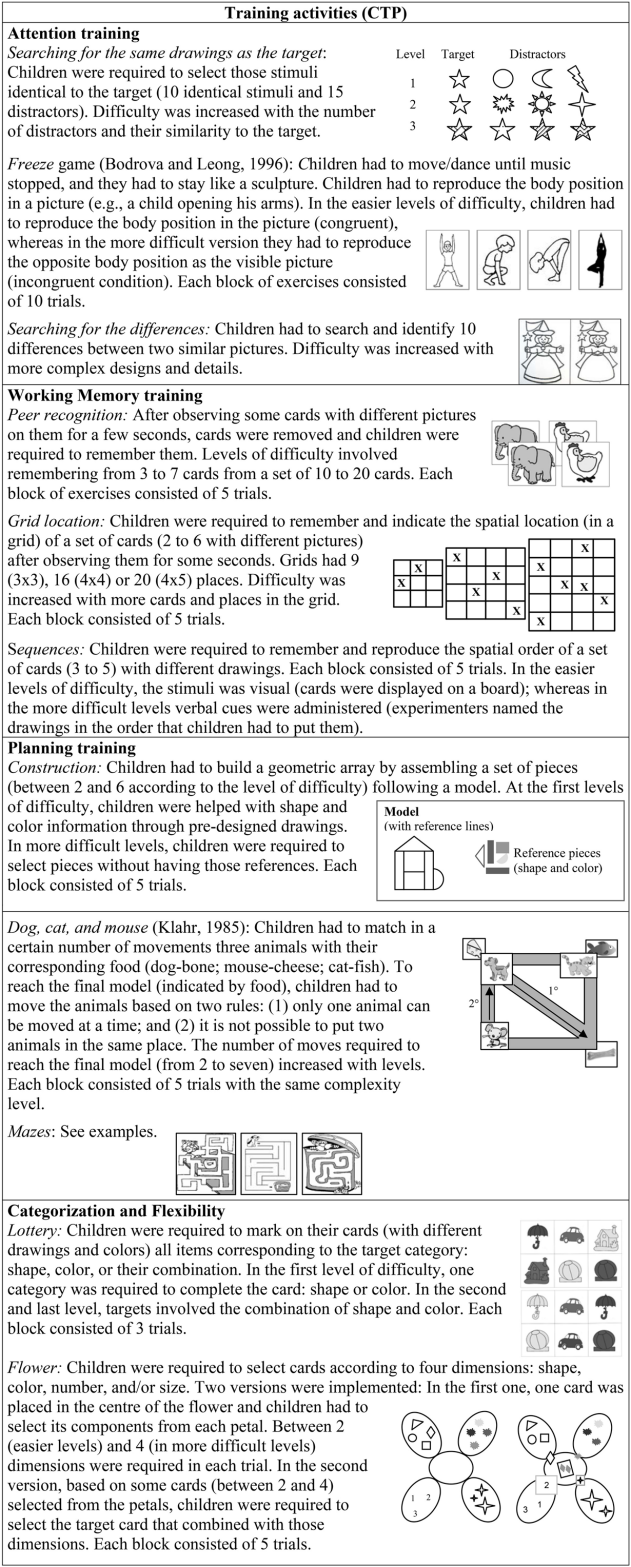
**Description of training activities in the CTP**.

In the CTP two modules were implemented, and put together with other activities developed by the government agency:

(1) The *cognitive training module* consisted of different activities demanding cognitive control, with increasing complexity. Activities for both training modalities were designed with a group of pedagogues (in this program activities differed from the basal and post-training cognitive assessment), and were implemented in weekly sessions (with two different activities within each session) during the school day, as an extracurricular classroom activity, for 16 weeks. As previously mentioned, for the present work only children in the group modality of training were considered in the analysis. Training groups were organized based on age and the maximum number of children necessary to form each group (from 10 to 25 children). Activities followed a scheme previously designed considering the cognitive demands and the time available for each session (30/40 min), and were implemented by two trainers (adults/children ratio = 1 per 10/15) (Figure 3).

The theoretical framework for the cognitive training module was the same one that was used in the SIP program. Also, the CTP applied the same session structure as the previous program (see Figure 2), but adapted to the group modality of training. That is, activities were solved with the participation of all children in the group, and only when 80% of children successfully solved 80% of trials in one block of exercises was the complexity level increased.

(2) The nutritional supplementation module was implemented for all children in the program, with the same frequency and methodology applied in the SIP.

Additionally, the government agency provided counseling for parents and adults working in the childcare centers. Researchers had no access to the information regarding these interventions.

### Measures in both programs

#### Socioeconomic, life, and health condition measures

In both programs data were collected during the school year (March to November) in a private interview with parents. A Socioeconomic Scale (NES) was used to evaluate parents' education and occupation levels, overcrowding, housing and sanitation conditions, to identify indicators of UBN (Boltvinik, 1995). Scores were assigned directly to mothers and fathers for educational and occupational backgrounds; however, only the higher score was considered for the total scores. For housing conditions, scores were assigned based on type of dwelling, floor, water, bathroom, ceiling, external walls, and home property. The Life Stressors and Social Resources Inventory (LISRES inventory) (Mikulic, 1999) was used to identify life stressors and social resources in the family. Specifically, this inventory measures sets of stressors and resources by administering two scales: (1) *stressors* that includes physical health dimensions (29 items), housing/neighborhood (22 items), finance (9 items), work (15 items), family (13 items), children (15 items), extended family (15 items), friends and social activities, and negative life events (12 items); and (2) *resources* that includes: finance (9 items), work (15 items), family (13 items), children (15 items), extended family (15 items), friends and social activities (15 items), and positive life events (10 items). The total score for each scale was calculated by adding scores obtained in each set of items, which were then transformed into *T*-scores (mean = 50, standard deviation = 10). Additionally, a set of questions concerning child peri- and post-natal health conditions was included in the interviews. Finally, in the SIP the Hamilton Scale (Hamilton, 1959, 1960) was employed to consider important aspects of mothers' mental health involved in self-regulation development (Buss et al., 2011). The scale consists of 14 items related to signs and symptoms of anxiety (7 items) and depression (7 items), which measures the intensity and frequency of such behaviors. The sum of the specific items for each type of sign results in a total score for depression and another for anxiety. There are no cut-points to distinguish subjects with and without anxiety or depression, so the results should be interpreted as a quantification of the intensity.

#### Cognitive measures

We evaluated cognitive performance in the pre- and post-training phases (Time 1 and Time 2), with a set of tasks administered by trainers in two sessions of about 40 min. Children were tested individually at their schools, in a quiet testing room. Testing was scheduled at times reported by teachers not to interfere with regular meals and activities. Examiners were blind to the objectives of the study and the composition of the groups.

In both programs, the following four tasks were used:

- The *Selective Attention* task—a manual adaptation of the computerized version of a subscale of the NEPSY battery (Korkman et al., 1998)—was used to evaluate attentional control. A set of sheets of paper with 25 pictures and one or more targets on each one was used. The child was required to identify and point to all the drawings that were identical to the target. Levels of difficulty (from 1 to 10) were determined according to the number of targets and the similarity between the target and the distractor drawings. Trials were administered until the child made more than 3 errors and/or omissions in three consecutive sheets. Scores represent the proportion of correct responses.
- The *Corsi Blocks* task (Berch et al., 1998) was used to assess visuo-spatial organization processes. In this task, a sequence of lights (from 2-to-8) was turned on (2 s each light, 1 s between lights). The child was required to remember and point to the boxes following the light sequences. Each level of difficulty comprised five trials with the same number of elements that children had to remember. Trials were administered until the child made three consecutive errors. A total score was computed as the sum of correct responses multiplied by level of difficulty (determined by the number of elements to remember on each trial).
- The *Tower of London* task (Shallice, 1982) was used to assess planning. In each trial the child was required to reach a goal configuration of three colored balls from a start configuration, moving one ball at a time, in a minimum number of movements. Any colored ball might be placed on top of any other ball, and children had to generate the appropriate action sequence. Levels of difficulty comprised exercises from 1 to 9 movements, and each one comprised five trials. Trials were administered until the child made three consecutive errors. A total score was computed as the sum of correct responses multiplied by level of difficulty (determined by the minimum number of movements necessary to reach the final model).
- The *Flexible Item Selection Task* (Jacques and Zelazo, 2001) was administered in the CPT, and in the second and third year of the SIP—which explains the reduction of the sample size—to assess abstract processing and cognitive flexibility. A set of cards was used conformed to items according to four dimensions: shape, color, size, and number. Tasks consisted of 12 trials in which the child was required to select two cards sharing the same dimension. The child was required to make two selections: first, select two cards equivalent in one way, and then select another two cards, equivalent in a different way. A total score was computed by adding correct responses in the first and second selection.

In addition, in the SIP, the *Stroop-like Day-Night* task was administered to assess inhibitory control processes (Gerstadt et al., 1994). The task consisted of 16 trials in which children were asked to say the opposite of what they saw in a series of cards. When a picture of a *sun* was presented, they had to say “night,” and when the picture showed a *moon*, they had to say “day.” A total score was computed as the sum of correct responses divided by the total number of trials.

## Results

### SIP program

Based on the literature, a set of variables were pre-selected as potential predictors of cognitive performance at baseline and of the change in performance between pre- and post-intervention: *housing conditions, overcrowding, parental education, parental occupation, mother's physical health, housing stressors, economic stressors, working stressors, couple stressors, child stressors, family stressors, friends, and social life stressors, negative life events, economic resources, working resources, couple resources, child resources, family resources*, *friends, and social life resources, positive life events, child health records, child age, child gender*, and *frequency of training sessions* (Sameroff et al., 1993; Brooks-Gunn and Duncan, 1997; McLoyd, 1998; Burchinal et al., 2000; Bradley and Corwyn, 2002; Gassman-Pines and Yoshikawa, 2006). Descriptive statistics for each study group are presented in Tables 2, 3.

**Table 2:** **Sociodemographic information of the SIP sample by group (continuous variables)**.

**Characteristic**	**Intervention**			**Control**				
	***n***	**Mean**	***SE***	***n***	**Mean**	***SE***	F^*^	P^*^
Child age (at baseline)	161	4.50	0.07	161	4.53	0.06	0.096	0.757
**SOCIOECONOMIC INFORMATION^a^**								
Parent education level^b^	169	6.08^c^	0.21	167	6.04^c^	0.19	0.002	0.961
Parent occupation background^b^	169	2.92^d^	0.17	170	2.89^d^	0.17	0.003	0.955
Housing^e^	166	8.64	0.14	167	8.95	0.14	2.288	0.131
Overcrowding conditions^f^	168	5.65	0.18	169	6.07	0.16	3.591	0.059
**LIFE STRESSORS AND SOCIAL RESOURCES^g^**								
Physical health	125	−49.38	0.88	129	−50.22	0.84	0.479	0.489
Housing stressors	127	−60.14	1.13	131	−58.79	1.12	0.728	0.394
Economic stressors	127	−65.71	0.63	131	−64.17	0.76	2.442	0.119
Working stressors	68	−48.40	0.79	69	−50.06	1.04	1.606	0.207
Couple stressors	103	−55.77	0.90	108	−56.35	0.84	0.227	0.634
Child stressors	125	−67.89	0.92	131	−66.96	0.85	0.548	0.460
Family stressors	112	−46.99	0.84	116	−47.68	1.00	0.276	0.600
Friends and social life stressors	107	−46.55	0.94	108	−45.52	0.83	0.682	0.410
Negative life events	127	−55.36	1.08	130	−54.49	1.05	0.331	0.565
Economic resources	126	38.36	0.07	130	38.50	0.09	1.484	0.224
Working resources	68	50.54	0.38	72	50.03	0.44	0.767	0.383
Couple resources	103	55.14	0.61	107	54.97	0.63	0.035	0.851
Child resources	125	65.95	0.47	130	65.94	0.52	0.000	0.985
Family resources	112	49.39	0.71	117	47.86	0.79	2.072	0.151
Friends and social life resources	105	49.96	0.98	107	47.28	1.23	2.879	0.091
Positive life events	127	48.77	0.77	130	50.74	0.77	3.274	0.072

aSocioeconomic information was obtained for most cases (this is the reason for the higher sample sizes in those variables).

b Highest educational and occupational levels reached by parents.

c Incomplete secondary school level.

d Non-skilled worker.

e Scale range: 3–12 points, with higher scores for better housing conditions.

f Scale range: 0–9 points, with higher scores for better conditions.

g T-scores from each item evaluated in the Life Stressors and Social Resources Inventory (LISRES).

* Univariate ANOVA was performed for each variable.

**Table 3:** **Sociodemographic information of the SIP sample by group (categorical variables)**.

**Characteristic**	**Intervention (*n* = 170)**		**Control (*n* = 173)**	
	***n***	**%**	***n***	**%**
**CHILD GENDER**				
Male	87	51.18	90	52.02
Female	83	48.82	83	47.98
**HEALTH HISTORY**				
With history of medical illness^a^	5	2.94	3	1.73
Without history of medical illness	165	97.06	170	98.27
**FREQUENCY OF TRAINING SESSION**				
Once a week	139	81.80	120	69.40
Twice a week	31	18.20	53	30.60

a Low weight at birth, premature, neurological, and/or perinatal disorders.

In order to identify basal differences between groups (intervention/control), univariate ANOVA models were applied with the pre-selected variables as dependent (separate analysis for each variable), *group* (intervention/control) as the fixed factor; and *age*, *gender* and *socioeconomic group* (UBN/SBN) as covariables. Results showed no significant differences between intervention and control groups, for all the socioenvironmental pre-selected variables (Table 2).

We then evaluated the assumptions for mixed models procedures, including residual normality, homocedasticity and independence. For this purpose, descriptive and univariate analyses, histograms and plot graphics as well as Levene tests were executed for each variable. All dependent variables showed violations of at least one of the considered criteria, and therefore these variables were transformed for the analysis (using square root or arcsine transformations). Finally, for each dependent variable, scores were transformed to *z*-scores prior to their inclusion in the mixed model analyses. This was done to have a common metric to compare intervention outcome across the tasks. Means and standard deviations for each cognitive task are presented in Table 5. Regarding basal cognitive performance, univariate ANOVA models were executed for each dependent variable in order to compare basal performance between the study groups. Analysis included *group* (intervention/control) as the fixed factor; baseline performance variables of each task were the dependent variables (separate analysis for each cognitive process); and *age*, *gender*, and *socioeconomic group* (UBN/SBN) were the covariables. Results indicated that both study groups were homogeneous with respect to their basal cognitive performance (Table 4).

**Table 4:** **Performance by task, time of assessment, and group in the SIP**.

**Time**	**Task**	**Control**			**Intervention**				
		***n***	**Mean**	***SE***	***n***	**Mean**	***SE***	***F***	***p***
1 (pre-intervention)	Tower of London	144	18.59	1.58	141	16.52	1.42	1.09	0.298
	Corsi blocks	124	11.56	0.64	113	12.81	0.74	1.51	0.220
	FIST^*^	96	4.66	0.33	73	3.89	0.33	2.02	0.157
	Selective attention	119	0.50	0.03	112	0.52	0.03	0.09	0.765
	Day/night	148	0.75	0.02	147	0.76	0.02	1.11	0.292
2 (post-intervention)	Tower of London	121	26.71	1.71	130	43.17	1.71	56.75	0.000
	Corsi blocks	93	15.40	0.92	82	23.20	1.39	27.40	0.000
	FIST^*^	67	5.49	0.31	56	6.50	0.29	6.78	0.010
	Selective attention	95	0.63	0.03	83	0.77	0.02	23.45	0.000
	Day/night	122	0.83	0.02	121	0.88	0.02	4.73	0.031

Time, moment of cognitive assessment;

* this task was implemented in the second and third year of the program implementation (2003/2004).

Considering the sample sizes and the extensive number of pre-selected independent variables to enter as predictors, we decided to reduce them with different procedures including: principal component analysis (PCA) from a set of variables, and correlation analysis (see next section).

#### Selection of potential predictors

A PCA was executed for variables selected from the Socioeconomic Status Scale and the LISRES inventory (see section Socioeconomic, Life, and Health Condition Measures) (PCA with a *Promax* rotation). The criteria used for the selection of the final PCA model were Eigenvalues over 1.00; Kaiser Coefficients over 0.6, total value of the commonalities over 10 and value of the commonalities for each variable over 0.4. The application of this procedure resulted in the identification of six factors (Table 5): Factor 1 (Household economic status) involves *economic* and *housing stressors*, and *economic resources*; Factor 2 (Family context) concerns *couple* and *child stressors*, *negative life events*, and *couple resources*; Factor 3 (Socioeconomic status) comprises *parental education* and *parental occupation level*, *housing conditions*, and *overcrowding*; Factor 4 (Social resources) involves *child*, *family*, and *social resources*; Factor 5 (Ties support) concerns *social* and *family stressors*; and Factor 6 (Life events) involves *positive life events*. All these factors were incorporated into the data set, and for all of them, higher scores refer to better environmental conditions.

**Table 5:** **PCA results depicting variables associated with socioeconomic status and level of stressors and resources in the SIP**.

	**Household economic status**	**Family context**	**Socio-economic status**	**Social resources**	**Ties support**	**Life events**
Economic stressors^a^	**0.84**	0.00	0.03	−0.12	−0.06	0.24
Economic resources^a^	**0.82**	−0.11	0.02	−0.01	−0.01	0.08
Housing stressors^a^	**0.60**	0.09	−0.03	0.12	0.16	−0.30
Couple stressors^a^	−0.02	**0.79**	0.12	0.05	0.11	0.15
Couple resources^a^	0.18	**0.64**	−0.05	0.24	−0.21	0.06
Child stressors^a^	−0.27	**0.58**	−0.01	0.11	0.29	0.14
Negative life events^a^	0.13	**0.42**	0.22	−0.20	0.24	−**0.41**
Overcrowding^b^	−0.19	0.15	**0.79**	−0.13	−0.21	−0.03
Housing conditions^b^	0.12	−0.10	**0.67**	0.05	−0.05	−0.27
Parents occupation level^b^	0.18	0.22	**0.56**	0.05	−0.05	0.09
Parents education level^b^	0.11	−0.37	**0.47**	0.26	0.24	0.22
Child resources^a^	0.06	0.10	0.03	**0.72**	−0.04	−0.02
Social resources^a^	−0.17	0.00	0.11	**0.70**	0.06	0.04
Family resources^a^	0.05	0.21	−0.15	**0.67**	−0.09	−0.15
Family stressors^a^	0.06	0.04	−0.14	−0.01	**0.75**	−0.11
Social stressors^a^	−0.04	0.08	−0.07	−0.04	**0.68**	0.09
Positive life events^a^	0.17	0.24	−0.05	−0.10	0.04	**0.87**

n = 221.

a Variables from the LISRES inventory.

b Variables from the Socioeconomic Status Scale.

Bold text indicates variables loading on each factor. These six factors were the only ones with eigenvalues larger than 1 in the correlation matrix, and the Scree plot also suggested the six-factor solution.

We performed a Pearson Correlation analysis including all potential predictors (socio-environmental factors derived from the PCA, and other variables not included in the PCA: demographic information, child health records and training exposure information) to identify variables with significant and high associations (Pearson coefficient over 0.5, *p* < 0.05). In those cases, only one of the correlated variables was selected for the subsequent steps—the selection was made based on the reliability of measures. The degree of association among independent and dependent variables was separately analyzed. For both, dependent and independent variables results showed no significant associations between variables (Tables 6, 7).

**Table 6:** **Correlations for independent variables in the SIP**.

	**1**	**2**	**3**	**4**	**5**	**6**	**7**	**8**	**9**	**10**	**11**
1. Factor 1 (Household economic status)	–										
2. Factor 2 (Family context)	0.265^**^	–									
3. Factor 3 (Socioeconomic status)	0.272^**^	0.002	–								
4. Factor 4 (Social resources)	0.156^*^	0.106	0.093	–							
5. Factor 5 (Ties support)	0.047	0.072	0.023	0.046	–						
6. Factor 6 (Life events)	−0.05	−0.138^*^	0.103	0.06	−0.102	–					
7. Maternal stress for physical health problems	−0.038	0.055	−0.055	−0.038	0.133	0.057	–				
8. Child sex	−0.059	−0.109	−0.045	−0.048	0.022	−0.043	0.000	–			
9. Health records	0.075	0.046	0.165^*^	0.024	0.281^**^	−0.075	0.119^*^	−0.018	–		
10. Child age	−0.15	0.013	−0.142	0.161	−0.073	−0.073	0.014	0.06	−0.081	–	
11. Frequency of sessions	0.046	0.121	−0.04	0.046	0.118	0.054	0.06	0.067	0.06	0.045	–

* p < 0.05;

** p < 0.01.

**Table 7:** **Correlations for dependent variables in the SIP**.

	**1**	**2**	**3**	**4**	**5**
1. Planning	–				
2. Visuo-spatial organization	0.380^**^	–			
3. Cognitive flexibility	0.335^**^	0.338^**^	–		
4. Attentional control	0.425^**^	0.307^**^	0.375^**^	–	
5. Inhibitory control	0.155^**^	0.195^**^	0.208^**^	0.189^**^	–

** p < 0.01.

#### Final models for the prediction analysis

Regarding the methodological approaches to analyze how ecological factors (i.e., micro- and mesosystemic) affect development, one of the traditional methods is the analysis of variance for repeated measures. During the past decade, a number of analytical models that overcome some disadvantages of the previous models (e.g., the ability to handle missing data) have been implemented for this type of analysis. These models are known as general linear mixed models (GLMM) (Long and Pellegrini, 2003; Singer and Willett, 2003; Arnau and Balluerka, 2004; Ferrer et al., 2004; Arnau and Bono, 2008; Seltman, 2009). Based on that, we conducted a sequence of mixed model analyses to identify significant predictors associated with higher levels of cognitive performance pre-intervention and with more improvement in cognitive performance from pre- to post-intervention.

We first conducted mixed model analyses with a basal predictor (*time*) and the interaction between *time* and *group* (intervention and control), in order to identify differences at baseline performance and trajectories (training impact) between both groups. Results showed a significant effect of *time* (*Attention*: *B* = 0.916, *p* < 0.0001; *Working memory*: *B* = 1.076, *p* < 0.0001; *Inhibitory control*: *B* = 0.396, *p* = 0.0004; *Flexibility*: *B* = 0.899, *p* < 0.0001; and *Planning*: *B* = 1.219, *p* < 0.0001), which means that all children (intervention and control) increased their baseline performance on all tasks. Additionally, results evidenced significant effects of *group* for most of the dependent variables (*Attention*: *B* = −0.493, *p* = 0.00004; *Working memory*: *B* = −0.590, *p* < 0.0001; *Flexibility:* *B* = −0.569, *p* = 0.0069; and *Planning:* *B* = −0.750, *p* < 0.0001), which means that children in the intervention group demonstrated improved performance after training compared to children in the control group. In addition, no significant differences at baseline were identified between groups.

Second, independent variables were grouped into four blocks of information: (1) *living conditions at home, life stressors, and social resources* (including the six factors derived from the PCA analysis); (2) *demographic information* (*child age* and *gender*, and *maternal stress for physical health problems*); (3) *child health* (*health records*); and (4) *training exposure* (*frequency of sessions* and *group*). Analyses were executed separately for each block. The interactions between independent variables with *time* and *group* (intervention or control) were included in the models, in order to identify differences between both groups at baseline performance and cognitive trajectories after training.

Before the next step, we tested the missing completely at random (MARC) assumption for the independent variables included in the blocks, and the cognitive performance variables. The assumption was verified for the independent variables (*X*^2^ = 22.85, *p* = 0.196), but not for the dependent cognitive variables. However, we did not input cognitive data based on the notion that doing this could alter the slope of the trajectories.

For each block, mixed model analyses were executed several times, removing the non-significant variables each time. This procedure was repeated until only significant variables were included for each block for each given cognitive outcome (dependent variable). The purpose of this was to reduce the number of independent variables to generate a final model of prediction to detect significant variables associated with cognitive performance. In general, results from this step showed significant socio-environmental predictors for each dependent variable, and overall, they evidenced a similar pattern, and yet also some differences, between the cognitive control processes and programs. A summary of results from these analyses is available from the authors upon request.

We combined the significant variables detected from each block in the previous step and included them in a final model of predictors. Similarly to what we explained above, we executed mixed model analyses several times, removing every time the non-significant predictors. At the end of this procedure, we identified a set of significant predictors (final model). This step was also executed for each dependent variable.

Each step was performed to ensure that the final model adequately reflected predictors associated with levels of pre-intervention cognitive performance and with improvement in cognitive performance from pre- to post-intervention. For the number of comparisons (*attention* = 4, *workingmemory* = 5, *inhibitorycontrol* = 2, *flexibility* = 6, *planning* = 4), the Bonferroni correction was used for a 0.05 level of significance (the final values of *p* were: attention = 0.0125, *workingmemory* = 0.01, *inhibitorycontrol* = 0.025, *flexibility* = 0.0083, *planning* = 0.0125).

#### Variables associated to cognitive performance and trajectories (final models)

We selected predictors for a final model for each program based on the results from previous steps. Table 8 includes the final parameter estimates for each cognitive process, and show significant predictors of cognitive performance at Time 1 (baseline) and of intervention trajectories (difference between Time 2 and Time 1). It is important to point out that for the final models there was a reduction in the number of participants due to the lack of information for all the predictors for some children.

**Table 8:** **Results for the final model for each cognitive process in the SIP**.

**Dependent variable^a^**	**Parameters**	**Estimate (*SE*)**	**η^2^**
Attention (*n* = 329)	Intercept	−1.578 (0.157)^***^	–
	Time	0.930 (0.098)^***^	0.349
	Child age	0.564 (0.058)^***^	0.295
	Group (control)^*^time	−0.477 (0.135)^**^	0.060
Working memory (*n* = 138)	Intercept	−1.348 (0.226)^***^	–
	Time	0.810 (0.102)^***^	0.404
	Group (control)	−0.398 (0.136)^*^	0.078
	Child age	0.540 (0.087)^***^	0.278
	Social resources^*^time	0.202 (0.092)^*^	0.050
Inhibitory control (*n* = 382)	Intercept	−0.141 (0.141)^***^	–
	Time	0.334 (0.075)^***^	0.064
	Child age	0.348 (0.051)^***^	0.131
Flexibility (*n* = 329)	Intercept	−1.284 (0.223)^***^	–
	Time	1.920 (0.294)^***^	0.185
	Child age	0.516 (0.075)^***^	0.138
	Frequency (once a week)	−0.577 (0.103)^***^	0.163
	Group (control)^*^time	−0.550 (0.191)^*^	0.050
	Child age^*^time	−0.423 (0.109)^*^^*^	0.085
Planning (*n* = 329)	Intercept	−2.133 (0.116)^***^	–
	Time	1.192 (0.078)^***^	0.444
	Child age	0.667 (0.039)^***^	0.501
	Group (control)^*^time	−0.720 (0.111)^***^	0.130

Estimates from Proc Mixed using the Restricted Maximum Likelihood estimator.

a Dependent Variables = Z-scores; parameter estimate standard errors (SE) listed in parentheses.

* p < 0.05;

** p < 0.001;

*** p < 0.0001.

In the final model for *Attention* (Model = time, group, age, time^*^group; Pseudo *R*^2^ = 0.0522; *n* = 329), the main effect of *time* shows that children from both groups, on average, significantly increased their basal performance around one standard deviation after training (*B* = 0.930; *p* < 0.0001). In addition, results show that children in the intervention group had higher performance after training than children in the control group (*B* = −0.477, *p* = 0.0005). Results also show effects of child *age* on Time 1 (*B* = 0.564; *p* < 0.0001). This result indicates that performance at baseline was higher for older children.

In the final model for *Working memory* [Model = time, group, age, social resources (factor 4), time^*^social resources (factor 4); Pseudo *R*^2^ = 0.2055; *n* = 138], the main effect of *time* shows that children from both groups increased their basal performance around one standard deviation (*B* = 0.810; *p* < 0.0001). In this final model the interaction between *time* and *group* was not included (due to dropping out of the model as non-significant; thus, differences between groups in the cognitive trajectory were not evaluated). Despite that, it is important to consider that results from previous analytical steps (prior to the inclusion of the predictors) show significant differences in cognitive trajectories between groups (i.e., more improvement in the intervention group).

With respect to the prediction of trajectories, results show that for each point on the *social resources* score (which means the perception of more resources associated with family, children and friends) children increased 0.202 points between pre and post-intervention performance in this task (*p* = 0.0303). Results also show effects of child *age* (*B* = 0.540; *p* < 0.0001) and *group* (*B* = −0.398; *p* = 0.0042) at Time 1. This pattern of results suggests that performance on Time 1 was higher for older children, and that children in the control group had, on average, lower baseline performance than children assigned to the intervention group.

For the *Inhibitory control* variable, results from the final model (Model = *time*, *age*, Pseudo *R*^2^ = 0.0203; *n* = 382) show main effects of the two variables included in the model. With respect to the effect of *time*, results suggest that children in both groups, on average, increased their initial performance after the intervention (*B* = 0.334; *p* < 0.0001). As in the previous case, in this model the variable *group* was not included. Also, results from previous steps showed non-significant differences in cognitive trajectories between groups for this task (i.e., both groups had similar change in performance from pre- to post-test).

Regarding the effect of *age* (*B* = 0.348; *p* < 0.0001), results suggest that older children had higher scores on this task. Moreover, our results show that none of the socio-environmental variables were related to the change in inhibitory control from pre- to post-intervention assessment.

In the final model for *Flexibility* (Model = time, group, age, frequency of sessions, time^*^group, time^*^age; Pseudo *R*^2^ = 0.1728; *n* = 329), the main effect of *time* shows that children in both groups increased their initial performance around two standard deviations (*B* = −1.920; *p* < 0.0001). In addition, results show that children in the intervention group had a higher increase in their performance after training than the control group (*B* = −0.550; *p* = 0.0046). Likewise, change in flexibility after training was also associated with child *age* (*B* = −0.423; *p* = 0.0002), which suggests that older children had smaller increases in their performance after training. Results also show that older children had higher scores at baseline (*B* = 0.516; *p* < 0.0001), and children who were involved in the intervention with a frequency of one session per week had 0.577 lower scores than children who were involved in the intervention with a frequency of two times per week (*p* < 0.0001).

Finally, in the model for *Planning* (Model = time, group, age, time^*^group; Pseudo *R*^2^ = 0.1355; *n* = 329), the main effect of *time* shows that children from both groups increased their initial performance around one standard deviation (*B* = 1.192; *p* < 0.0001). Results also suggest significant effects of *group* on cognitive trajectories after training, such that children in the intervention group had higher scores after the intervention than children in the control group (*B* = 0.720; *p* < 0.0001). Also, our results indicate effects of child *age* (*B* = 0.667; *p* < 0.0001) on Time 1 performance, which suggest that scores tended to be higher for older children.

### CTP program

In this program we implemented the same procedures as in the SIP. First, a set of variables was pre-selected as potential predictors of cognitive performance at baseline and of the cognitive performance change after intervention: *housing conditions, overcrowding, parental education, parental occupation, mother's physical health, housing stressors, economic stressors, working stressors, couple stressors, child stressors, family stressors, friends and social life stressors, negative life events, economic resources, working resources, couple resources, child resources, family resources, friends and social life resources, positive life events, child health records, child age, child gender, family composition, reception of social benefits (subsidies), mother age, low weight at birth, premature, neurological disorders, perinatal disorders*, and the *number of training sessions*. Descriptive statistics are presented in Tables 9, 10.

**Table 9:** **Socio-demographic information of the CTP sample (continuous variables)**.

**Characteristic**	***n***	**Mean (*SD*)**
Child age (at baseline)	333	3.97 (0.73)
Mother age	318	29.05 (5.76)
**SOCIOECONOMIC INFORMATION^a^**		
Parent education level^b^	320	7.07^c^ (2.68)
Parent occupation background^b^	321	3.78^d^ (2.92)
Housing^e^	321	9.91 (2.16)
Overcrowding conditions^f^	321	6.26 (2.30)
**LIFE STRESSORS AND SOCIAL RESOURCES^g^**		
Physical health	268	−48.97 (10.71)
Housing stressors	268	−55.49 (10.99)
Economic stressors	268	−61.87 (8.08)
Working stressors	190	−49.67 (7.64)
Couple stressors	188	−54.82 (8.88)
Child stressors	257	−64.83 (8.79)
Family stressors	258	−46.37 (8.49)
Friends and social life stressors	236	−45.82 (8.17)
Negative life events	268	−59.57 (12.96)
Economic resources	266	38.42 (0.84)
Working resources	189	51.01 (2.57)
Couple resources	189	54.34 (6.37)
Child resources	256	66.39 (5.34)
Family resources	258	48.98 (7.43)
Friends and social life resources	232	46.91 (11.12)
Positive life events	268	55.38 (10.59)
Number of training sessions^h^	292	19.34 (5.96)

a Socioeconomic information was obtained in most cases (this is the reason for the higher sample sizes in those variables).

b Highest educational and occupational levels reached by parents.

c Incomplete secondary school level.

d Non-skilled worker.

e Scale range: 3–12 points, with higher scores for better housing conditions.

f Scale range: 0–9 points, with higher scores for better conditions.

g T scores from each item evaluated in the Life Stressors and Social Resources Inventory (LISRES).

h The total number of sessions vary between children due to their absence to the institutions.

**Table 10:** **Socio-demographic information of the CTP sample (categorical variables)**.

**Characteristic**	***n***	**%**
**CHILD SEX**		
Male	175	52.55
Female	158	47.45
**HEALTH HISTORY**		
With history of medical illness^a^	160	49.54
Without history of medical illness	163	50.46
**SINGLE PARENT HOUSEHOLD**		
Yes	123	38.44
No	197	61.56
**FAMILY WITH SOCIAL BENEFIT SUPPORT (SUBSIDIES)**		
Yes	169	52.65
No	152	47.35
**SOCIOECONOMIC GROUP**		
Unsatisfied basic need home	161	49.80
Satisfied basic need home	160	50.20

a Low weight at birth, premature, neurological, and/or perinatal disorders.

We adapted some of these variables for the analysis. Specifically, *age of mother* and *number of sessions* were re-categorized into categorical predictors for the analyses in order to have comparative groups within each predictor, and be able to distinguish between very young, young or older mothers as well as low, middle or high training exposure. In both cases, based on descriptive statistics, we created three groups [1 = values below 1 standard deviation (SD) of the mean; 2 = values between 1 *SD* above and below the mean, 3 = values above 1 *SD* of the mean]. Specifically, for *age of mother* groups were: less than 24 years, between 24 and 34.4 years, more than 34.4 years. For training exposure groups were: low training exposure (less than 13 sessions), middle training exposure (13–24 sessions), and high training exposure (more than 24 sessions).

Regarding the dependent variables, we evaluated the assumptions for mixed models procedures, and, like in the SIP, all dependent variables showed violations of at least one of the criteria considered. Therefore, these variables were transformed for the analysis (using square root or arcsine transformations). Finally, for each dependent variable, scores were transformed to z to have a common metric to be able to compare intervention outcome across the tasks. Means and standard deviations for each cognitive task are presented in Table 11.

**Table 11:** **Performance by task and time of assessment in the CTP**.

	**Time 1**			**Time 2**		
**Task**	***n***	**Mean**	***SD***	***n***	**Mean**	***SD***
Tower of London	284	11.61	15.53	271	21.79	21.52
Corsi blocks	280	8.71	6.85	270	10.83	6.77
FIST	280	15.80	8.87	271	18.42	7.63
Selective attention	273	0.31	0.24	271	0.42	0.25

Time 1, cognitive assessment pre-intervention (baseline); Time 2, cognitive assessment post-intervention.

In this program it was also necessary to reduce the number of pre-selected variables to enter them into the analyses, and the same procedures executed in the previous program were implemented (see next section).

#### Selection of potential predictors

First, a PCA was executed for variables from the Socioeconomic Status Scale and the LISRES inventory. Same criteria used for the SIP were considered for this program, and seven components were identified (see Table 12): Factor 1 (Housing conditions) involves *housing conditions*, *overcrowding*, and *housing stressors*; Factor 2 (Economic status) contains *economic stressors* and *economic resources* variables; Factor 3 (Ties support) comprises *family stressors*, *negative life events* and *child stressors*; Factor 4 (Social aspects of health) concerns *social stressors*, *physical health*, and *social benefit reception*; Factor 5 (Social resources) involves *family, child* and *social resources* variables; Factor 6 (Family composition) comprises *family composition* and *parental occupation level*; and Factor 7 (Positive events) concerns the variable *positive life events*. All these factors were incorporated into the data set, and for all of them, higher scores refer to better environmental conditions.

**Table 12:** **PCA results depicting variables associated with socioeconomic status and level of stressors and resources in the CTP**.

	**Housing conditions**	**Economic status**	**Ties support**	**Social aspects of health**	**Social resources**	**Family composition**	**Positive events**
Housing stressors^a^	**0.83**	−0.06	0.26	0.03	−0.09	0.03	0.18
Housing conditions^b^	**0.79**	−0.05	−0.20	0.10	−0.02	0.04	−0.16
Overcrowding^b^	**0.72**	0.11	0.02	−0.29	−0.04	−0.10	0.03
Economic stressors^a^	−0.02	**0.75**	−0.03	0.18	−0.03	0.24	0.20
Economic resources^a^	0.02	**0.71**	−0.09	0.05	0.11	0.01	−0.12
Family stressors^a^	−0.01	0.01	**0.79**	0.19	−0.01	−0.12	0.05
Negative life events^a^	−0.06	0.60	**0.59**	−0.14	−0.13	−0.01	−0.23
Child stressors^a^	0.10	−0.25	**0.58**	0.26	0.10	0.09	−0.04
Social stressors^a^	−0.11	−0.03	0.18	**0.72**	−0.08	0.05	−0.04
Physical health^a^	0.00	0.24	0.16	**0.68**	−0.05	−0.02	0.14
Social benefits reception^b^	0.20	0.29	−0.08	**0.41**	0.19	−0.13	−0.35
Social resources^a^	−0.11	0.14	−0.19	−0.04	**0.76**	−0.16	0.15
Child resources^a^	−0.04	−0.22	0.19	−0.07	**0.66**	0.30	−0.16
Family resources^a^	0.02	0.12	0.54	−0.03	**0.62**	−0.04	0.09
Family composition ^b^	−0.09	0.08	−0.04	0.10	−0.09	**0.88**	−0.01
Parents occupation level^b^	0.20	0.29	−0.11	−0.16	0.15	**0.63**	0.02
Positive life events^a^	0.05	−0.01	−0.03	0.07	0.09	−0.02	**0.93**

n = 256.

a Variables from the LISRES inventory.

b Variables from the Socioeconomic Status Scale.

Bold text indicates variables loading on each factor. These seven factors were the only ones with eigenvalues larger than 1 in the correlation matrix, and the Scree plot also suggested the seven-factor solution.

We performed a Pearson Correlation analysis including socio-environmental factors derived from the PCA and other variables (demographic information, child health records, and training exposure information). For the independent variables, results show a pair of variables with a moderately high degree of association: *Low weight at birth* and *Premature* (*r* = 0.53, *p* < 0.0001) (Table 13). Based on these results, low weight at birth was selected for the block analysis, as it can result from preterm birth or intrauterine growth restriction, or a combination of both (Shah and Ohlsson, 2002). Results show no significant correlations for the dependent variables (Table 14).

**Table 13:** **Correlations for independent variables in the CTP**.

		**1**	**2**	**3**	**4**	**5**	**6**	**7**	**8**	**9**	**10**	**11**	**12**	**13**	**14**	**15**	**16**
1.	Factor 1	–															
2.	Factor 2	0.359^**^	–														
3.	Factor 3	−0.120	−0.043	–													
4.	Factor 4	0.043	0.043	0.083	–												
5.	Factor 5	0.270^**^	0.108	−0.099	0.012	–											
6.	Factor 6	0.150^*^	0.036	0.164^*^	0.012	0.156^*^	–										
7.	Factor 7	−0.092	−0.112	0.075	0.039	−0.131	−0.030	–									
8.	Child sex	−0.015	−0.023	−0.094	0.035	−0.040	−0.051	−0.029	–								
9.	Child age	−0.056	0.006	−0.001	0.058	−0.048	0.034	0.000	−0.067	–							
10.	Parental education	0.373^**^	0.310^**^	−0.209^**^	−0.105	0.145^*^	0.129	−0.037	0.038	−0.070	–						
11.	Low weight at birth	0.042	−0.081	−0.168^*^	−0.103	0.069	0.011	−0.044	−0.029	−0.031	0.059	–					
12.	Neurological disorders	−0.006	−0.120	−0.079	−0.031	0.121	−0.008	0.035	0.030	0.073	−0.003	0.240^**^	–				
13.	Perinatal disorders	−0.054	−0.181^**^	−0.026	−0.171^*^	0.035	0.122	−0.145^*^	0.028	−0.034	0.000	0.075	0.206^**^	–			
14.	Premature	−0.023	−0.090	−0.155^*^	−0.076	0.015	0.104	−0.034	0.045	−0.023	0.037	0.529^**^	0.230^**^	0.189^**^	–		
15.	Training exposure	−0.009	0.125	0.023	−0.105	−0.030	0.015	−0.051	0.006	0.047	0.030	0.025	−0.081	−0.054	0.037	–	
16.	Mother age	0.076	0.050	0.082	−0.087	0.023	0.021	−0.030	−0.044	0.102	0.007	0.029	0.026	0.054	0.053	0.175^**^	–

* p < 0.05;

** p < 0.01.

**Table 14:** **Correlations for dependent variables in the CTP**.

	**1**	**2**	**3**	**4**
1. Planning	–			
2. Visuo-spatial organization	0.244^**^	–		
3. Cognitive flexibility	0.408^**^	0.208^**^	–	
4. Attentional control	0.476^**^	0.289^**^	0.486^**^	–

** p < 0.01.

#### Creation of the final models for the prediction analysis

With the purpose of identifying significant predictors for both, basal cognitive performance and cognitive performance change between pre- to post-intervention, we conducted a sequence of mixed model analyses. We first ran a model with a basal predictor (*time*). Results showed significant estimates for all variables (*Attention*: *B* = 0.4596, *p* < 0.0001; *Working memory*: *B* = 0.3816, *p* < 0.0001; *Flexibility*: *B* = 0.3458, *p* < 0.0001; *Planning*: *B* = 0.5745, *p* < 0.0001), which means that children improved their performance on all these tasks following the group modality of cognitive training.

Second, independent variables were grouped into four blocks of information: (1) *Living conditions at home, life stressors and social resources* (including the seven factors derived from the PCA analysis); (2) *demographic information* (*child age* and *gender*, *parental education* and *mother age group*); (3) *child health* (*low weight at birth*, *neurological disorders*, *perinatal disorders*); and (4) *training exposure* (*training exposure group*). Analyses were executed separately for each block. The model included the interaction between each variable with *time* (see section Study Design, Participants, and Procedures).

Before the next step, the MARC assumption was tested for the independent variables included in the blocks, and the cognitive performance variables. The assumption was verified for all variables (independent variables: *X*^2^ = 4.46, *p* = 0.216; basal cognitive performance variables: *X*^2^ = 0.931, *p* = 0.818; post-intervention cognitive performance variables: *X*^2^ = 0.513, *p* = 0.916).

For each block, we executed mixed model analyses several times, removing the non-significant variables each time. In general, results from this step showed significant socio-environmental predictors for each dependent variable, and overall, they evidenced a similar pattern, and yet also some differences between cognitive processes. As was mentioned for the SIP, the summary of results of these analyses is available upon request.

Significant variables from the previous step were combined and included in a final model of prediction (also for this step, analyses were executed several times removing every time the non-significant predictors). At the end of this procedure we identified a set of significant predictors (final model). For the number of comparisons (*attention* = 7, *workingmemory* = 3, *flexibility* = 5, *planning* = 6), the Bonferroni correction was used for a 0.05 level of significance (the final values of *p* were: *attention* = 0.00714, *workingmemory* = 0.01667, *flexibility* = 0.01, *planning* = 0.0083).

#### variables associated to cognitive performance and trajectories (final models)

Table 15 includes the final parameter estimates for each cognitive process, and shows significant predictors of cognitive performance at Time 1 (baseline) and of intervention trajectories (difference between Time 2 and Time 1).

**Table 15:** **Results for the final model for each cognitive process in the CTP**.

**Dependent variable^a^**	**Parameters**	**Estimate (*SE*)**	**η^2^**
Attention (*N* = 188)	Intercept	−1.621 (0.199)^***^	–
	Family composition	0.179 (0.051)^**^	0.063
	Child age	0.607 (0.070)^***^	0.294
	Housing conditions^*^time	0.191 (0.063)^**^	0.050
	Middle training exposure^*^time	0.495 (0.214)^*^	0.036
Working memory (*N* = 215)	Intercept	−1.429 (0.138)^***^	–
	Time	0.403 (0.083)^***^	0.105
	Ties support	0.120 (0.047)^*^	0.032
	Child age	0.600 (0.064)^***^	0.307
Flexibility (*N* = 188)	Intercept	−1.655 (0.197)^***^	–
	Time	0.364 (0.076)^***^	0.115
	Housing conditions	0.139 (0.054)^*^	0.037
	Family composition	0.172 (0.054)^**^	0.055
	Child age	0.579 (0.073)^***^	0.266
	Middle training exposure	0.333 (0.161)^*^	0.030
	High training exposure	0.430 (0.185)^*^	0.030
Planning (*N* = 188)	Intercept	−1.771 (0.197)^***^	–
	Child age	0.765 (0.071)^***^	0.395
	Family composition^*^time	0.150 (0.060)^*^	0.035
	High training exposure^*^time	0.564 (0.225)^*^	0.033

Estimates from Proc Mixed using the Restricted Maximum Likelihood estimator.

a Dependent variables = Z-scores; parameter estimate standard errors (SE) listed in parentheses.

* p < 0.05;

** p < 0.01;

*** p < 0.0001.

In the final model for Attention (Model = time, housing conditions, family composition, child age, training exposure, housing conditions^*^time, training exposure^*^time; Pseudo *R*^2^ = 0.0953, *n* = 188), the main effect of time (after controlling for the other variables in the model) became non-significant (*B* = 0.064; *p* = 0.7463). However, results suggest significant effects of housing conditions (*B* = 0.191; *p* = 0.003) and marginally significant effects of training exposure (middle exposure: *B* = 0.4952; *p* = 0.0217) on cognitive trajectories. This also suggests that changes in performance from pre- to post-assessment enhanced with increasing scores on housing conditions (higher scores in this factor indicate better housing conditions, less perception of stress associated with housing, and less overcrowding conditions), and with a middle exposure to training in comparison to a low exposure. Results also show main effects of family composition (*B* = 0.1791; *p* = 0.0006) and child age (*B* = 0.6067; *p* < 0.0001) at Time 1. This pattern of results suggests that performance on the attention task was enhanced with increasing scores on family composition (higher scores for this factor indicate better parental occupation backgrounds and the presence of two parents in the home), and for older children.

In the final model for *Working memory* (Model = *time, ties support* and *child age*; Pseudo *R*^2^ = 0.0817, *n* = 215), the main effect of *time* shows that children, on average, significantly increased their basal working memory performance 0.40 of a standard deviation from pre- to post-test performance (*p* < 0.0001). None of the socio-environmental variables were related to performance change in working memory between assessments. Results also show effects of *ties support* (*B* = 0.1199; *p* = 0.0113) and *child age* (*B* = 0.5992; *p* < 0.0001) on Time 1 performance. This pattern of results indicates that performance in working memory at Time 1 was higher in older children and with increasing scores on the *ties support* factor (higher scores for this factor are associated with the perception of less stress associated with the family and the children, and with less negative life events).

In the final model for *Flexibility* (Model = *time, housing conditions, family composition, child age*, and *training exposure*; Pseudo *R*^2^ = −0.0098, *n* = 188), the main effect of *time* shows that children, on average, significantly increased their basal performance by 0.3641 points after training (*p* < 0.0001). None of the socio-environmental variables were related to the performance change in flexibility from pre- to post-assessment. Results also suggest significant effects of *family composition* (*B* = 0.1717; *p* = 0.0016), *child age* (*B* = 0.5790; *p* < 0.0001), and marginally significant effects of *housing conditions* (*B* = 0.1386; *p* = 0.0113) and *training exposure* (middle exposure: *B* = 0.3329, *p* = 0.0402; high exposure: *B* = 0.4304, *p* = 0.0209) at Time 1. This pattern of results indicates that performance at Time 1 was higher for older children; with increasing scores on *housing conditions* (higher scores on this factor indicate better housing conditions, less perception of stress associated with housing, and less overcrowding conditions) and *family composition* (higher scores for this factor indicate better parental occupation backgrounds and the presence of two parents at home); and with high or middle exposure to training activities.

In the final model for *Planning* (Model = time, family composition, child age, training exposure, family composition^*^time, training exposure^*^time; Pseudo *R*^2^ = 0.0105, *n* = 188), the main effect of *time* (after controlling for the other variables in the model) became non-significant (*B* = 0.2634; *p* = 0.1613). However, results suggest significant effects of *family composition* (*B* = 0.1495; *p* = 0.0130) and marginally significant effects of *training exposure* (high exposure: *B* = 0.5643; *p* = 0.0131) on cognitive trajectories. These results suggest that change between pre- and post-training performances increases with increasing scores on *family composition* (higher scores for this factor indicate better parental occupation backgrounds and the presence of two parents at home); and with high exposure to training activities. Results also show main effects of *child age* (*B* = 0.7653; *p* < 0.0001) on Time 1, which suggests that baseline performance in the planning task was higher in older children.

### Basal performance comparisons between SIP and CTP

We executed univariate ANOVA models for common variables between both programs (variables for the Socioeconomic Status Scale, the Lisres inventory and performance in attentional control, visuo spatial organization and planning), in order to compare basal cognitive performance and socio-environmental factors. The model included *program* (SIP/CTP) as the fixed factor; baseline cognitive performance and socio-demographic variables were the dependent variables (analyses were run separately for each variable); and *age*, *gender*, and *socioeconomic group* (UBN/SBN) were the covariables. Comparisons between the two programs regarding socioeconomic status and life conditions evidence significant differences between programs in some variables: overcrowding conditions [*F*_(1−658)_ = 17.83; *p* < 0.0001], economic resources [*F*_(1−522)_ = 5.14; *p* = 0.024], couple resources [*F*_(1−399)_ = 4.19; *p* = 0.041], friends and social life resources [*F*_(1−444)_ = 5.55; *p* = 0.019], positive life events [*F*_(1−525)_ = 41.85; *p* < 0.0001], child stressors [*F*_(1−513)_ = 12.58; *p* < 0.0001], family stressors [*F*_(1−486)_ = 5.50; *p* = 0.019], and negative life events [*F*_(1−525)_ = 13.67; *p* < 0.0001]. In the other variables (housing conditions, parent education and occupation levels, mother physical health, housing stressors, economic stressors, working stressors and resources, couple stressors, friends and social life stressors, working resources, child resources, and family resources) no significant differences were found between programs. With respect to cognitive performance at Time 1, results show significant differences in attentional control [*F*_(1−500)_ = 20.68; *p* < 0.0001] and visuo-spatial organization [*F*_(1−513)_ = 4.45; *p* = 0.035]. Also, results show marginally significant differences between programs in planning basal performance [*F*_(1−565)_ = 3.17; *p* = 0.076].

## Discussion

The main goals of the present study were to investigate: (1) how socio-environmental factors influence baseline cognitive performance; and (2) the influence of environmental factors on cognitive trajectories (based on pre- and post-intervention assessments of attention, memory, inhibitory control, flexibility and planning). We analyzed data from two intervention programs implemented in Argentina for such objectives. Both programs have their strengths and weaknesses: the SIP included a control group, and the cognitive training module consisted of an exercising approach—same materials, different trials—, whereas the CTP did not include a control group, but the cognitive training module included pedagogic activities. Despite these advantages and limitations, results allow identifying significant predictors of both basal cognitive performance and performance changes between cognitive assessments.

Although most of the socio-environmental factors considered in the present study have often been found to be related to cognitive functioning (e.g., Brooks-Gunn and Duncan, 1997; Burchinal et al., 2000; Bradley and Corwyn, 2002; Evans, 2004; Gassman-Pines and Yoshikawa, 2006; Rhoades et al., 2011; Sarsour et al., 2011), they have rarely been simultaneously considered in training studies, so their effect on training outcome has been unclear.

Results across both intervention studies show that baseline performance of healthy preschoolers on a set of basic cognitive processes (attention, working memory, inhibitory control, flexibility, and planning), and their trajectories after training and exercising (based on pre- and post-intervention assessments) can be modulated by specific socio-environmental and individual factors. Specifically, for all cognitive processes in both programs, older children had higher baseline performance. Additionally, different variables were identified as influencing performance at baseline on attention, working memory, flexibility, and planning. Specifically, in the CTP, for attention, results show that children from dual-parent households and parents with better occupational backgrounds had higher performance at baseline. The same was verified for flexibility, where in addition, performance was higher at baseline among children with better housing conditions, as well as those who had more training sessions. Finally, in the case of working memory, our results show that baseline performance was higher for children living in homes with more ties support.

This pattern of results is in agreement with the literature on the impact of poverty on cognitive performance, suggesting that worse environmental conditions (i.e., *housing conditions, parental occupation level, family composition, social resources*) predict lower cognitive performance (e.g., Conger and Brent-Donnellan, 2007; Hackman and Farah, 2009; Lipina and Colombo, 2009; Hackman et al., 2010; Rhoades et al., 2011). In addition, results could indicate a differential sensitivity of each cognitive process to different socio-environmental factors. To investigate and examine this differential sensitivity to context, similar studies with other tasks for the same processes, as well as with samples of a wider age range (from infancy through adolescence), should be implemented. In general, the literature about poverty and cognitive development is based on a broad definition of poverty. In that sense, the identification of differential sensitivity of cognitive control processes to some environmental factors would be important to the design of interventions aimed at improving cognitive performance (Lipina et al., 2011).

With respect to cognitive trajectories from pre- to post-training, different profiles were also identified for each cognitive process and intervention program. In the SIP, for attention, working memory, flexibility, and planning, training impacts were verified (children in the intervention group had more improvement than children in the control group). Additionally, trajectories for the same tasks were predicted by some environmental factors and program characteristics. Specifically, in the case of flexibility, child *age* predicted the trajectory (older children had lower change in performance from pre- to post-test). A different pattern was verified for working memory trajectories, in which the variable *social resources* was a marginally significant predictor of change (performance change increased for children from homes with more social resources).

Results in the CTP show that *housing conditions* scores predicted the attention trajectory, indicating that change in performance from pre- to post assessment was higher for children with better home conditions, less overcrowding and fewer housing stressors. A different pattern was verified for planning trajectories, where *family composition* was the significant predictor of change. That is, change in planning performance from pre- to post-test was higher for children living in homes with two parents and with better parental occupation levels. Additionally, for both tasks (i.e., attention and planning), *training exposure* was also a marginally significant predictor of change in performance, indicating that children with more training sessions tended to have higher performances after training.

It is important to note that in the CTP, the design did not include a control group because governmental agencies did not allow researchers to do that. Nevertheless, taking into account results obtained in the SIP, designed as a randomized control program, results for the CTP show similar trends regarding the associations between better socio-environmental and individual factors (e.g., age, housing conditions, and family composition) and higher cognitive performance. Specifically, in both programs children improved their basal performance in attention, working memory, flexibility and planning. Results also suggest, for the same dependent variables that older children present larger performance increments. Additionally, for working memory, children with more family and child resources tended to perform better at baseline (CTP) or had higher post-training improvements (SIP).

Again, results for the trajectories were not homogeneous across dependent variables and programs. These variations are consistent with other studies that indicate that not all aspects of deprivation or intervention impacts affect the relation between cognitive performance and socioeconomic status (e.g., Hoff, 2006). These differences, both between cognitive performances and between programs, could suggest differential susceptibilities of each cognitive process, as well as different patterns of cognitive integration throughout development (Garon et al., 2008).

In the present study, it is important to consider that children attending both programs had different socio-demographic characteristics, and also had different cognitive performance in the same tasks at baseline stages. Furthermore, considering the absence of significant socio-environmental predictors in both programs for some baseline performances (e.g., planning in the SIP and CTP) and for cognitive trajectories (e.g., working memory, and flexibility in the CTP; and inhibitory control in the SIP), it is necessary to consider in future analyses other socio-environmental variables that could be related to those particular cognitive processes. Additionally, it would be important to consider the administration of other cognitive measures for the same processes (Lyons and Zelazo, 2011; Bauer and Zelazo, 2013). In spite of that, it is possible to conceive that not all cognitive skills are equally susceptible to training (Jolles and Crone, 2012; Rueda et al., 2012). Therefore, although we did not verify significant predictors for some baseline performances and for some cognitive trajectories in both programs, based on the present analyses we cannot conclude that socio-environmental conditions would not predict them.

Likewise, it is important to consider differences between sample characteristics when interpreting differences in results between programs and cognitive processes [e.g., intervention programs contexts of application (prekindergarten or child cares), staff instruction, supervision and curriculum design among childcare centers in CPT; districts of implementation; and PCA results] (Ramey and Ramey, 2003; Barnett, 2011; Reynolds et al., 2011; Weiland and Yoshikawa, 2012).

Besides the need to deepen the analysis of different sensitivity to the context of cognitive processes (both, baseline performances and the effects of interventions), studies with different cognitive measures, socio-environmental variables, different levels of analysis-such as individual susceptibility or sensitive periods (Thomas and Johnson, 2008; Obradović et al., 2010), would contribute in such a sense.

Finally, it is important to mention that results must be tempered by some study limitations. First, each of the cognitive processes was measured using a single task. Future studies should consider using a variety of tasks that target the same cognitive process. Second, the analysis of change of performance over time by training is based on two time points of measurement. Although short time effects can be evaluated, future studies should include the analysis of long-term effects of training for a better understanding of the links between family and child background and training impact. Third, it is possible that the analyses in this study were underpowered, perhaps partially due to the number of evaluated subjects. Despite that, results tend to be similar to those of other studies that have used socio-environmental factors as well as training exposure to predict cognitive development (e.g., Ramey and Ramey, 2003; Rhoades et al., 2011). Finally, with respect to the program design, the CTP did not include a control group as the aim was to compare two training modalities (individual/group). Although results showed similar profiles in both programs, studies should include control designs.

Overall, this work contributes to elucidating the complex relationships between socio-environmental factors, cognitive development and intervention strategies, suggesting that environmental factors could be associated in particular ways with performance in tasks demanding attention, working memory, inhibitory control, cognitive flexibility and planning.

## Conclusions

Analysis suggests that environmental factors moderated cognitive performance at baseline and through the course of the interventions in some, but not all, cognitive processes.

In sum, the contribution of the present study consists in the identification of factors that contribute to performance changes after cognitive interventions. The methodology implemented gives additional information about the impact of training, traditionally evaluated by comparing pre and post mean scores. It also contributes to the current literature about the emergence and development of cognitive processes, and their modulation by interventions in longitudinal analyses. The implemented approach and results are important for informing future intervention designs for both children and their families in Argentina.

## Author contributions

M. Soledad Segretin participation as operator and supervisor in both training programs; tabulation and preparation of datasets; statistical analysis design and execution; manuscript writing. Sebastián J. Lipina participation as designer and coordinator in both intervention programs; statistical analysis design and supervision; manuscript writing. M. Julia Hermida collaboration in preparation of datasets; manuscript review. Tiffany D. Sheffield statistical analysis execution and supervision; manuscript review. Jennifer M. Nelson statistical analysis design and supervision; manuscript review. Kimberly A. Espy statistical analysis design and supervision; manuscript review. Jorge A. Colombo participation as designer in both intervention programs; manuscript review.

### Conflict of interest statement

The authors declare that the research was conducted in the absence of any commercial or financial relationships that could be construed as a potential conflict of interest.
